# Indomethacin Treatment Post-irradiation Improves Mouse Parotid Salivary Gland Function via Modulation of Prostaglandin E_2_ Signaling

**DOI:** 10.3389/fbioe.2021.697671

**Published:** 2021-07-21

**Authors:** Kristy E. Gilman, Jean M. Camden, Lucas T. Woods, Gary A. Weisman, Kirsten H. Limesand

**Affiliations:** ^1^Department of Nutritional Sciences, University of Arizona, Tucson, AZ, United States; ^2^Department of Biochemistry and Christopher S. Bond Life Sciences Center, University of Missouri, Columbia, MO, United States

**Keywords:** radiation, head and neck cancer, salivary glands, xerostomia, prostaglandin E_2_, indomethacin, immunomodulation, regeneration

## Abstract

Annually, >600,000 new cases of head and neck cancer (HNC) are diagnosed worldwide with primary treatment being surgery and radiotherapy. During ionizing radiation (IR) treatment of HNC, healthy salivary glands are collaterally damaged, leading to loss of function that severely diminishes the quality of life for patients due to increased health complications, including oral infections and sores, cavities, and malnutrition, among others. Therapies for salivary hypofunction are ineffective and largely palliative, indicating a need for further research to uncover effective approaches to prevent or restore loss of salivary gland function following radiotherapy. Previous work in our lab implicated prostaglandin E_2_ (PGE_2_) as an inflammatory mediator whose release from radiation-exposed cells promotes salivary gland damage and loss of function. Deletion of the P2X7 purinergic receptor for extracellular ATP reduces PGE_2_ secretion in irradiated primary parotid gland cells, and salivary gland function is enhanced in irradiated P2X7R^–/–^ mice compared to wild-type mice. However, the role of PGE_2_ signaling in irradiated salivary glands is unclear and understanding the mechanism of PGE_2_ action is a goal of this study. Results show that treatment of irradiated mice with the non-steroidal anti-inflammatory drug (NSAID) indomethacin, which reduces PGE_2_ production via inhibition of cyclooxygenase-1 (COX-1), improves salivary gland function compared to irradiated vehicle-treated mice. To define the signaling pathway whereby PGE_2_ induces salivary gland dysfunction, primary parotid gland cells treated with PGE_2_ have increased c-Jun N-terminal Kinase (JNK) activation and cell proliferation and reduced amylase levels and store-operated calcium entry (SOCE). The *in vivo* effects of blocking PGE_2_ production were also examined and irradiated mice receiving indomethacin injections have reduced JNK activity at 8 days post-irradiation and reduced proliferation and increased amylase levels at day 30, as compared to irradiated mice without indomethacin. Combined, these data suggest a mechanism whereby irradiation-induced PGE_2_ signaling to JNK blocks critical steps in saliva secretion manifested by a decrease in the quality (diminished amylase) and quantity (loss of calcium channel activity) of saliva, that can be restored with indomethacin. These findings encourage further attempts evaluating indomethacin as a viable therapeutic option to prevent damage to salivary glands caused by irradiation of HNC in humans.

## Introduction

Each year, >600,000 new cases of head and neck cancer (HNC) are diagnosed across the world (Johnson et al., 2020). Effective approaches to treat HNC include surgical excision of the tumor followed by IR, with or without chemotherapy (Cramer et al., 2019). During radiation treatment, salivary glands, located proximal to tumors, are collaterally damaged leading to reduced salivary gland function. Reduced saliva output causes numerous health complications, including increased rates of oral infections, cavities, and malnutrition and an overall poorer quality of life (Grundmann et al., 2009). Current treatment options for salivary hypofunction are palliative, relatively ineffective and costly to patients, indicating a need for further research to improve the quality of life for HNC patients (Jensen et al., 2019).

Wound healing is a complex regenerative process that occurs in three sequential but overlapping phases following tissue damage: (i) hemostasis/inflammation, (ii) proliferation/re-epithelialization, and (iii) remodeling. While each phase of the response is essential for adequate wound repair, dysregulation at any stage can lead to insufficient repair or chronic inflammation and excessive scarring. Hemostasis/inflammatory responses begin immediately following a wounding event and typically last for about 3 days (Reinke and Sorg, 2012). Radiation-induced inflammatory responses have been studied in salivary glands but present conflicting results (Jasmer et al., 2020). Research suggests that interleukin (IL)-6 mediates induction of cellular senescence in irradiated (13 Gy) submandibular glands (SMGs), with elevated levels seen at 3 h and 14 days post-IR. However, both IL-6 knockdown and IL-6 treatment prior to radiation exposure unexpectedly protected SMGs from senescence at 8 weeks post-IR, leaving the role of IL-6 during the inflammatory response to radiation difficult to understand (Marmary et al., 2016). Interestingly, treatment of SMGs with an adenovirus containing the neurotrophic factor, neurturin, prevents hypofunction in irradiated (6 Gy fractions × 5 days) salivary glands, when given pre- but not post-IR (Lombaert et al., 2020). Radiation treatment also resulted in a significant increase in inflammation-associated gene expression in irradiated mice (300 days post-IR) and minipigs (16 weeks post-IR) that was reduced by neurturin-expressing adenovirus administration prior to radiation exposure and was associated with normalized morphology and increased size and function of the salivary gland (Lombaert et al., 2020). In contrast, another study demonstrated that there is a decrease in immune-related gene expression and reduced macrophage numbers in irradiated (15 Gy) SMGs at days 7–28 post-IR, whereas adenoviral-induced activation of the Sonic hedgehog (Shh) pathway at day 3 post-IR increased immune gene expression and macrophage numbers (Zhao et al., 2020). These studies suggest that an effective therapy to prevent salivary gland dysfunction due to radiation should target the hemostasis/inflammation phase of tissue damage, i.e., 0–3 days post-IR.

The second phase of the wound healing response, cell proliferation, is necessary to replace cells lost following damage; however, the homeostatic regulation of proliferation and differentiation is necessary to promote functional tissue repair. It has previously been proposed that the proliferative phase encompasses days 3–21 of the wound healing process, with transition to the remodeling phase occurring from day 21 through 1 year post-damage (Reinke and Sorg, 2012). In irradiated salivary glands, it has been shown that compensatory proliferation begins at day 5 post-IR and is mediated by activation of c-Jun N-terminal kinase (JNK) signaling (Wong et al., 2019). However, cell proliferation rates in irradiated salivary glands remain elevated compared to non-irradiated glands at chronic timepoints, days 30, 60, and 90 post-IR (Grundmann et al., 2010). Despite the increase in cell proliferation rates in irradiated salivary glands, the cells remain undifferentiated, as indicated by decreased amylase levels (Grundmann et al., 2010; Hill et al., 2014; Morgan-Bathke et al., 2014). Various pharmacological agents have been evaluated for restoration of irradiated parotid glands, where reducing the proliferative response correlates with increased salivary gland function *in vivo* (Grundmann et al., 2010; Hill et al., 2014; Morgan-Bathke et al., 2014). These data suggest that dysregulated signaling during the transition from the proliferative to remodeling phases of the wound healing process should be targeted to enhance salivary gland function following radiation damage.

The P2X7 receptor (P2X7R) for extracellular ATP released from damaged cells is a component of the innate immune system. Previous work from our lab showed that deletion or pharmacological antagonism of the P2X7R prevents salivary gland dysfunction in mice caused by radiation exposure (Gilman et al., 2019). Interestingly, mouse primary parotid gland cells lacking the P2X7R secrete significantly lower levels of the biologically active lipid, prostaglandin E_2_ (PGE_2_), basally and following radiation exposure, which suggests that P2X7R-mediated PGE_2_ release may lead to salivary gland dysfunction that could be reversed by blocking the P2X7R (Gilman et al., 2019). PGE_2_ is produced from plasma membrane phospholipid-derived arachidonic acid that is first converted to prostaglandin H_2_ by cyclooxygenases (COXs), COX-1 and COX-2, and then to PGE_2_ by microsomal PGE synthase-1 (mPGES-1), mPGES-2 or cytosolic PGE synthase (cPGES) (Gilman and Limesand, 2020). PGE_2_ acts by binding to four different E-prostanoid receptors (EPRs), EP1-4R, to induce the activation of multiple G proteins (Gilman and Limesand, 2020). EPRs, typically EP2R and EP4R, also can transactivate the epidermal growth factor receptor (EGFR) (Jiang et al., 2017). Based on their ability to activate G protein and EGFR signaling, PGE_2_-bound EPRs regulate multiple physiological processes, including, proliferation, differentiation, survival, cytokine production, immune cell migration and vasodilation/vasoconstriction (Dennis and Norris, 2015; Gilman and Limesand, 2020). PGE_2_ also induces the phosphorylation of intracellular c-Jun terminal kinase (JNK) and it’s downstream target c-Jun (Zeng et al., 2015; Zhong et al., 2015), which we have previously shown mediates the induction of compensatory proliferation in irradiated salivary glands (Wong et al., 2019). Indomethacin is a non-steroidal anti-inflammatory drug (NSAID) that functions via nonselective and reversible inhibition of COXs to reduce the synthesis of eicosanoids, including PGE_2_ (Lucas, 2016). Due to the postulated role of PGE_2_ in the induction of radiation-induced salivary gland dysfunction, we evaluated the hypothesis that indomethacin treatment would restore irradiated salivary gland function by blocking PGE_2_ production and subsequent activation of JNK-mediated compensatory proliferation.

## Materials and Methods

### Mice

Animals were maintained according to the University of Arizona Institutional Animal Care and Use Committee (IACUC) regulations with protocols approved by the IACUC. Four to eight week-old C57BL/6J (stock no. 000664) or FVB/NJ mice (stock no. 001800) were purchased from Jackson Labs (Bar Harbor, ME, United States). Age- and sex-matched mice of the same genotype were randomly assigned to treatment groups. Mice were on 12 h light/dark cycles and housed in vented cages with food and water *ad libitum*. Where indicated, mice received intraperitoneal (IP) injections of vehicle (sterile saline with 10% ethanol) or indomethacin (1 mg/kg body weight, Sigma, no. I7378, St. Louis, MO, United States) prepared from a 10 mg/mL stock solution in 100% ethanol, warmed at 55°C for 5 min, diluted to 1 mg/mL in saline and sterilized by passage through a 0.22 μm Polyvinylidene difluoride (PVDF) filter.

### Radiation Treatment

Mice were sedated via an IP injection of a mixture of ketamine/xylazine (50–10 mg/kg), constrained in 50 mL tubes and shielded with >6 mm lead, leaving only the head and neck region exposed. Mice or cells were placed in the radiation field and received a single 5 Gy dose of radiation from a ^60^Co Teletherapy unit at 80 cm distance from the source and ∼0.3–0.4 Gy/min (Theatron-80, Atomic Energy of Canada, Ottawa, ON, Canada) or a 225 kV X-ray unit at 48 cm distance from the source and 1.4 Gy/min (RS2000, Rad Source Technologies, Buford, GA, United States).

### Saliva Collection

Salivary flow rates were evaluated on days 3, 10, or 30 following radiation. Saliva production was stimulated with an IP injection of carbachol (0.25 mg/kg body weight) and whole saliva was collected for 5 min via vacuum aspiration into pre-weighed tubes and then snap-frozen. Salivary flow was calculated by taking the difference in tube weight (post-collection minus pre-collection) and dividing by 5 min to express saliva secretion in milligrams per minute. Saliva flow rates were normalized to the average of the non-irradiated, vehicle-injected group on each day of collection.

### Primary Cell Preparation

Parotid glands were removed from four to eight week-old C57BL/6J mice, prepared as previously described (Gilman et al., 2019) and suspended in primary cell culture media: DMEM/F12 containing (in wt/vol, except where noted) epidermal growth factor (0.4%; Fisher Scientific), insulin (0.125%; Invitrogen), glutamine (1.25%; Invitrogen), nonessential amino acids (1%; Invitrogen), transferrin (0.125%, Invitrogen), retinoic acid (0.05%; Sigma-Aldrich), trace elements (1%; Thermo Fisher Scientific), gentamycin (0.5%; Thermo Fisher Scientific), fungizone (0.2%; Invitrogen), hydrocortisone (0.04%; Sigma-Aldrich), and fetal bovine serum (10% vol/vol; Thermo Fisher Scientific, unless the use of serum-free media was indicated). Cells were used immediately as dispersed parotid cell aggregates or seeded onto 35 mm collagen-coated plates and grown for 2 days prior to use. Primary parotid cells or aggregates from independent preparations are considered as a single replicate for assays and at least three replicates were performed from separate preparations for all experiments.

### Cyclooxygenase Activity Assay

Primary cells were prepared as described above. On day 2 of culture, cells were treated with indomethacin (25 μM in saline from the 100% ethanol stock solution) or vehicle (saline with 0.1% ethanol) and cells were collected 24 h later. Protein was extracted as described below and COX activity was measured via a COX activity assay (Abcam, no. ab204699, Cambridge, United Kingdom) following the manufacturer’s instructions.

### Prostaglandin E_2_ Enzyme-Linked Immunosorbent Assay

Primary cells were prepared as described above. On day 2 of culture, media was replaced and cells were treated with indomethacin (25 μM in saline) or vehicle (saline with 0.1% ethanol) 1 h prior to receiving a 5 Gy dose of radiation. Cell-free supernatants were collected at indicated timepoints following radiation and the PGE_2_ concentrations were determined with an enzyme-linked immunosorbent assay (PGE_2_ ELISA, R&D Systems, no. KGE004B, Minneapolis, MN) following the manufacturer’s instructions, where PGE_2_ was normalized to the protein concentration of the corresponding cell culture dish.

### Prostaglandin E_2_ Treatment

Vehicle (DMSO) or varying doses (10 nM, 100 nM, 1 μM, 10 μM) of PGE_2_ (Cayman Chemical, no. 14010, Ann Arbor, MI, United States) were diluted in serum-free primary cell media and used to treat dispersed parotid cell aggregates at the indicated timepoints for dose-response and kinetic analyses. For amylase staining, PGE_2_ was diluted to 10 μM in serum-complete primary cell media and cells were treated on culture plates for 24 h prior to collection. Cells were centrifuged and resuspended in lysis buffer to extract proteins as described below.

### Protein Isolation and Quantification

Parotid glands were harvested and snap-frozen from untreated and irradiated mice at day 8 or 30 post-IR. Tissues were homogenized in radioimmunoprecipitation assay (RIPA) buffer, with protease inhibitor cocktail (30 μL/mL; Sigma, no. P8340), sodium orthovanadate (5 mM) and phenylmethylsulfonyl fluoride (PMSF, 1 mM). Tissue homogenates were incubated on ice for 30 min, sonicated for 1–2 min and centrifuged at 12,000 RPM for 10 min at 4°C to remove cell debris. Cells or aggregates were collected by scraping, centrifuged, resuspended in tissue protein extraction reagent (T-PER, Thermo no. 78510) with protease inhibitor cocktail (30 μL/mL), sodium orthovanadate (5 mM), and PMSF (1 mM), incubated on ice for 10–15 min and centrifuged at 12,000 rpm for 10 min at 4°C to remove cell debris. Protein content was measured with the Pierce Coomassie Plus Bradford assay (Thermo, no. 23236, Waltham, MA, United States) or the Bicinchoninic acid (BCA) assay (Thermo, no. 23225).

### Immunoblotting

Five to fifty micrograms of protein were added to 2X Laemmli sample buffer and boiled for 5–10 min at 95–100°C. Sample proteins were separated via electrophoresis on 10% polyacrylamide gels. Proteins were transferred to PVDF membranes (Millipore, no. IPVH00010) at 100 volts for 1 h. Membranes were blocked in either 5% (w/v) nonfat milk or 5% (w/v) bovine serum albumin (BSA, Fisher Bioreagents, no. BP1600-100) dissolved in tris-buffered saline with Tween 20 (TBS-T, 20 mM Tris base, 137 mM NaCl, 0.05% Tween 20 (v/v), pH 7.6) for 1 h at room temperature, washed in TBS-T three times for 5 min each and incubated in primary antibody overnight at 4°C. The following rabbit-anti-mouse primary antibodies were used: phospho-JNK/SAPK (p54/p46)^T183/Y185^ (1:1,000, Cell Signaling, no. 4668, Danvers, MA, United States), JNK/SAPK (p54/p46, 1:1,000, Cell Signaling, no. 9252) phospho-c-Jun^*S*73^ (1:1,000, Abcam, no. ab30620), c-Jun (1:1,000, Cell Signaling, no. 9165), amylase (1:3,000 in 3% BSA, Sigma, no. A8273), Aquaporin 5 (AQP5; 1:1,000, Millipore-Sigma, no. AB3559-50UL), muscle, intestine and stomach expression 1 (MIST1; 1:1,000, Cell Signaling, no. 14896), EP1 (1:500; Cayman Chemical no. 101740), EP2 (1:200; Cayman Chemical no. 101750), EP3 (1:200; Cayman Chemical no. 101760), EP4 (1:200; Cayman Chemical no. 101775), ERK1/2 (1:1,000; Cell Signaling, no. 9102) or β-tubulin (1:1,000, Cell Signaling, no. 2128). Membranes were washed three times for 5 min each in TBS-T then incubated in HRP-conjugated goat anti-rabbit IgG antibody (1:10,000 in 5% nonfat milk TBS-T; Cell Signaling, no. 7074S) for 1 h at room temperature. Membranes were washed three times for 5 min each in TBS-T then incubated with Pierce ECL Western Blotting Substrate (Thermo, no. 32109) for 1 min or SuperSignal West Pico Plus Chemiluminescent Substrate (Thermo, no. 34577) for 5 min. Membranes were exposed to autoradiography film (Genesee, no. 30-810) and developed using an Srx-101A X-ray film processor (Konica). Membranes were stripped using Restore Western Blotting Stripping Buffer (Fisher, no. 21063), blocked in 5% nonfat milk and re-probed for loading controls. Densitometry was performed using ImageJ software (NIH).

### 5-Ethynyl-2′-Deoxyuridine Incorporation Assay

Primary parotid gland cells were prepared as described above and were cultured on 18 mm collagen-coated glass coverslips (Neuvitro, no. H-18-collagen, Vancouver, WA, United States). On day 4 of culture, cells were treated with vehicle (DMSO), PGE_2_ (Cayman Chemical, no. 14010), or EP receptor-selective (EP1R-EP4R) agonists at indicated doses: 17-phenyl trinor PGE_2_ (EP1R agonist; Cayman Chemical, no. 14810), AH13205 (EP2R agonist; Santa Cruz Biotechnology, no. sc-214513), sulprostone (EP3R agonist; Cayman Chemical, no. 14765) and CAY10598 (EP4R agonist; Cayman Chemical, no. 13281). Cells were serum-starved for 2 h prior to treatment. All compounds were solubilized in DMSO, mixed into serum-free primary cell culture media (described above) and cells were treated for 24 h. During the last hour of EPR agonist treatment, cells were incubated with 5-ethynyl-2’-deoxyuridine (EdU) from the Click iT EdU cell proliferation kit (Invitrogen, no. 10337). EdU was fluorescently labeled following the manufacturer’s instructions. Coverslips were mounted on glass slides with Prolong Gold Antifade Mountant (Thermo Fisher Scientific, no. 36934). Images were captured on a Leica DM5500 fluorescence microscope (Leica Microsystems, Wetzlar, Germany) with 4-megapixel Pursuit camera (Diagnostic Instruments, Inc.) at 200× magnification. EdU positive cells and total cells were manually counted. Groups were quantified by averaging the number of positive cells out of the total number of cells from 5 fields of view/slide and 3 slides per treatment. Graphs depict the average number of positive cells/total number of cells. Each symbol represents an independent sample.

### Intracellular Calcium Quantification

Intracellular free Ca^2+^ concentration ([Ca^2+^]_*i*_) in isolated parotid epithelial cells was quantified as previously described (Woods et al., 2018). Briefly, primary parotid gland cells untreated or treated with 10 μM PGE_2_ for 24 h in serum-free DMEM/F12 media containing gentamycin (50 μg/ml) were washed with assay buffer (120 mM NaCl, 4 mM KCl, 1.2 mM KH_2_PO_4_, 1.2 mM MgSO_4_, 1 mM CaCl_2_, 10 mM glucose, 15 mM HEPES, 1% (w/v) BSA, pH 7.4) and then adhered to chambered coverslips using Cell-Tak cell adhesive (Corning Inc., Corning, NY, United States) and loaded with 2 μM of the calcium indicating dye, fura-2-AM (Life Technologies, Carlsbad, CA, United States) in assay buffer for 30 min at 37°C, washed and incubated in dye-free assay buffer for 30 min. Prior to use, cells were washed again and placed in calcium-free assay buffer containing 0.2 mM EDTA and baseline fluorescence values were collected for 60 s prior to stimulation with carbachol (100 μM) for 210 s to evaluate muscarinic type 3 receptor functionality. Then, 3 mM calcium was added for the final 90 s to quantitate store-operated calcium entry (SOCE) into cells. Changes in the 340/380 nm fluorescence excitation ratio (505 nm emission) were detected using an InCyt Dual-Wavelength Fluorescence Imaging System (Intracellular Imaging, Cincinnati, OH, United States). Resulting fluorescence ratios were converted to [Ca^2+^]_*i*_ (nM) using a standard curve created with solutions containing known concentrations of Ca^2+^.

### Histology

Salivary glands were harvested from mice at indicated timepoints post-IR and submerged in 10% neutral buffered formalin overnight. Tissues were sent to IDEXX Bioanalytics (Columbia, MO, United States) for processing where they were dehydrated with ethanol and xylene, embedded in paraffin and sectioned at 4 μm.

### Immunofluorescence

Salivary gland sections were incubated at 37°C for 20 min, submerged in Histoclear (National Diagnostics, no. HS-200) for 10 min and rehydrated in ethanol gradations (100, 95, 70, and 50%) and deionized water for 10 min each. Sections were permeabilized for 15 min in 0.2% (v/v) Triton X-100 in PBS, then washed with PBS three times for 5 min each. Next, antigen retrieval was completed by microwaving sections in citric acid (pH 6.0) for 10 min and then cooling for 20 min. Sections were washed with PBS three times for 5 min each, blocked in 0.5% New England nuclear blocking agent (Perkin Elmer, no. 2346249) for 1 h at room temperature and then incubated in anti-amylase antibody (1:1,000 in 1% BSA; Sigma, no. A8273) or anti-Ki67 antibody [1:400 in 1% BSA, Cell Signaling, no. 9129] overnight at 4°C. Slides were washed three times for 10 min each, incubated in fluorophore-conjugated goat anti-rabbit IgG antibody (Alexa Fluor 488, 1:500 in 1% BSA; Thermo, no. A-11008) for 1 h at room temperature, washed again three times for 10 min each and then rinsed in water for 5 min. Cell nuclei were stained with 4’,6-diamidino-2-phenylindole (DAPI, 1 μg/mL; Invitrogen, no. D1306) for 3 min then washed with water for 10 min. Amylase-stained sections were mounted with 50% glycerol in 10 mM Tris–HCl (pH 8.0), and Ki67-stained sections were mounted with ProLong Gold Antifade mounting agent (Invitrogen, no. P36934) and imaged the following day. Images were captured on a Leica DM5500 with 4-megapixel Pursuit camera at 400× magnification with identical camera settings used for all images. Quantification was done using ImageJ software with images from 20 fields of view per mouse with 5 mice per treatment for amylase area, or 5 fields of view per mouse with 4 mice per treatment for Ki67. Graphs depict the mean percentage of amylase positive area or the average percentage of Ki67 positive cells out of the total cell number. Each point represents an independent mouse.

### Statistical Analysis

Statistical tests were run using GraphPad Prism 9 software (San Diego, CA, United States). Normally distributed data was assessed by Brown-Forsythe test. To determine significance between groups, a student’s *t*-test, or a one-way analysis of variance (ANOVA) was used, followed by Dunnett’s *post hoc* comparisons when comparing to a control group, or Bonferroni’s *post hoc* comparisons when comparing all groups. A *p*-value of less than 0.05 is considered statistically significant. Specific *p*-values are indicated by the number of asterisks above groups (^∗^*p* < 0.05, ^∗∗^*p* < 0.01, ^∗∗∗^*p* < 0.001, ^****^p < 0.0001).

## Results

### Post-radiation Indomethacin Treatment Restores Salivary Gland Function in Mice

**FIGURE 1:**
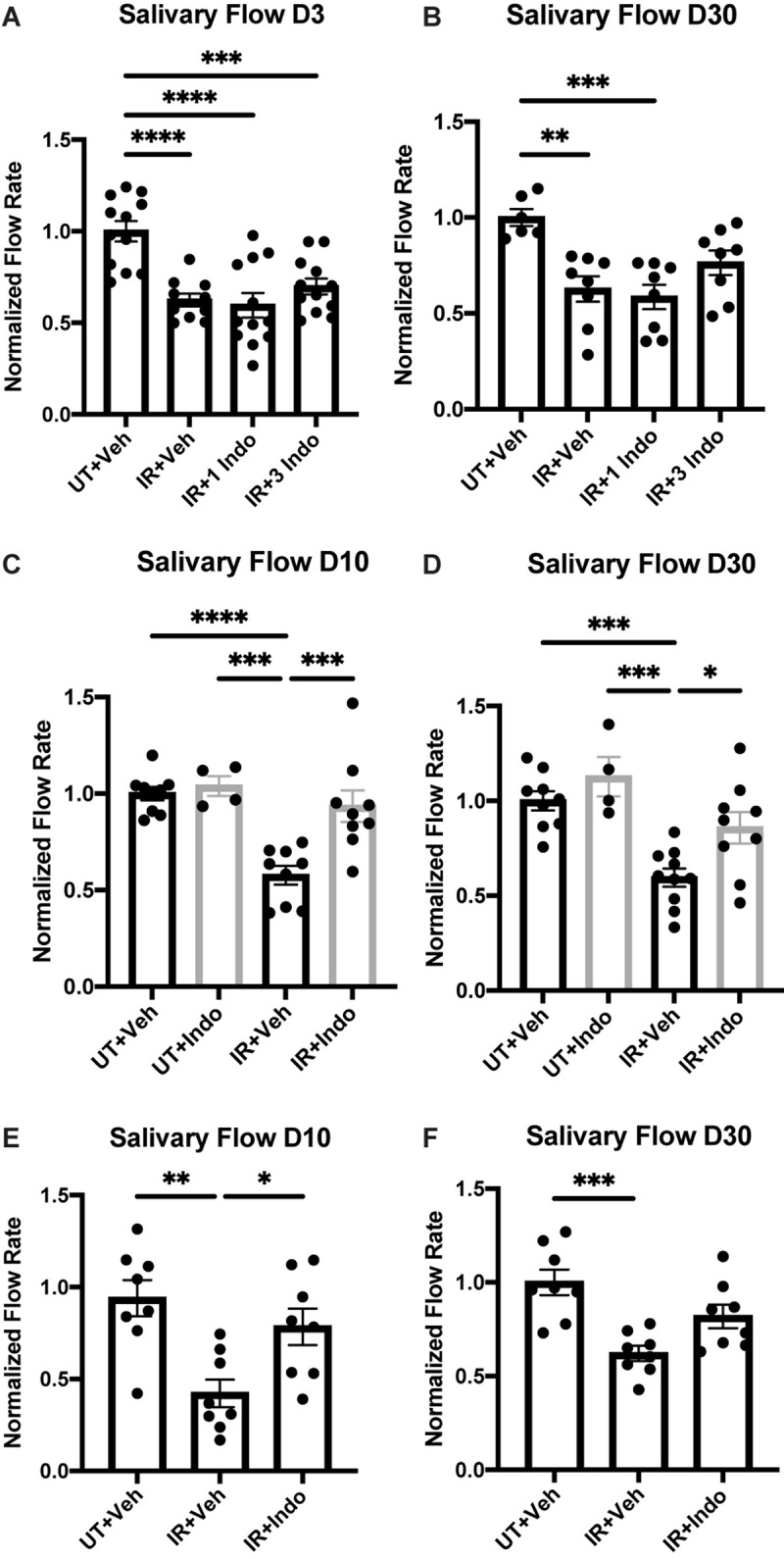
Pre- versus post-radiotherapy indomethacin treatment differentially modulates salivary flow rates in mice. **(A–D)** FVB or **(E,F)** C57BL/6J mice were untreated (UT) or treated with 5 Gy ionizing radiation (IR) and intraperitoneal injections of vehicle (Veh, saline with 10% ethanol) or indomethacin (Indo, 1 mg/kg body weight) **(A,B)** 1 h prior to radiation (1 Indo) or again at days 1 and 2 post-IR (3 Indo) or **(C–F)** at days 3, 5 and 7 post-IR. **(A–F)** Carbachol-stimulated (0.25 mg/kg body weight) saliva was collected on **(A)** day 3, **(C,E)** day 10 and **(B,D,F)** day 30 post-IR, as described in section “Materials and Methods.” Graphs represent the mean ± standard error of the mean (SEM). Each closed circle represents an independent sample. Significant differences were determined via a one-way ANOVA followed by Bonferroni’s *post hoc* comparisons (*p < 0.05, **p < 0.01, ***p < 0.001, ****p < 0.0001). Groups with no asterisks are not significantly different from each other.

FVB/NJ (Figures 1A–D) or C57BL/6J (Figures 1E,F) mice were exposed to 5 Gy of radiation and were injected with the NSAID, indomethacin (1 mg/kg body weight), 1 h prior to IR only (Figures 1A,B, IR+1 Indo) or again at days 1 and 2 post-IR (Figures 1A,B, IR+3 Indo). Alternatively, mice received injections of indomethacin at days 3, 5, and 7 post-IR (Figures 1C–F). Stimulated salivary flow rates were measured on day 3, representative of an acute timepoint post-IR damage, and day 30, representative of a chronic timepoint post-IR damage (Grundmann et al., 2010). The results indicate that a single injection of indomethacin (1 Indo) prior to IR exposure does not preserve salivary gland function post-IR (Figures 1A,B). Interestingly, mice receiving both pre- and post-IR indomethacin (3 Indo) injections have reduced stimulated saliva output on day 3 that is only modestly improved by day 30 (Figures 1A,B). In contrast, FVB/NJ (Figures 1C,D) and C57BL/6 (Figures 1E,F) mice receiving indomethacin injections on days 3, 5, and 7 post-IR (Figures 1C–F) show salivary flow rates at days 10 and 30 post-IR similar to untreated, vehicle injected (UT+Veh) mice (Figures 1C,D). In addition, indomethacin treatment alone does not alter salivary gland function in mice not treated with IR (Figures 1C,D). Combined, these data suggest that inhibiting eicosanoid synthesis with indomethacin following radiation exposure leads to restoration of salivary gland function.

### Indomethacin Treatment of Primary Parotid Gland Cells Reduces COX-1 Activity and Prostaglandin E_2_ Secretion Induced by Radiation

**FIGURE 2:**
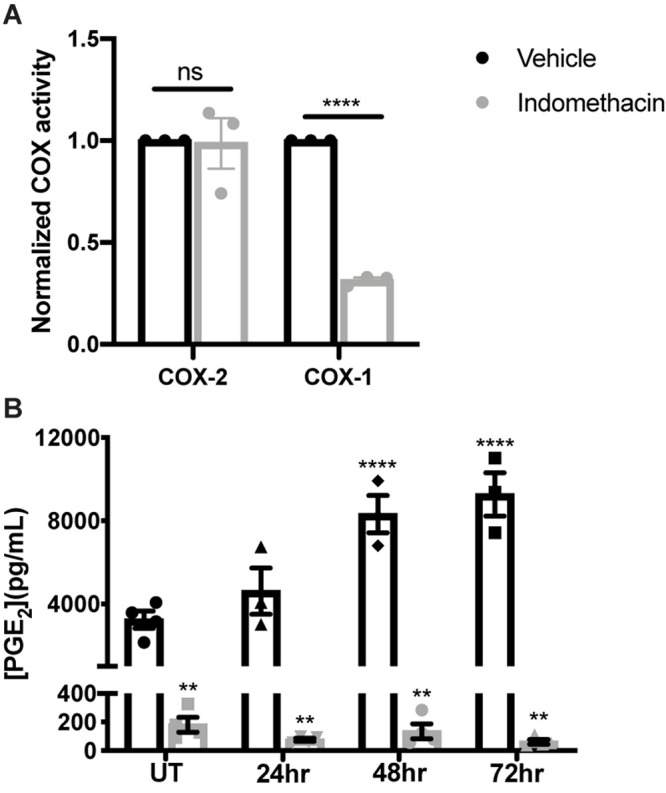
Indomethacin treatment reduces cyclooxygenase-1 (COX) function and PGE_2_ release in irradiated primary parotid acinar cells. Primary parotid acinar cells were prepared from C57BL/6J mice as described in section “Materials and Methods” and were untreated (UT) or treated with 5 Gy radiation (IR) with vehicle (Veh, saline with 0.1% ethanol) or indomethacin (Indo, 25 μM) for 1 h prior to IR. **(A)** Untreated cells were collected after 24 h of indomethacin treatment, protein was extracted and COX activities were measured with a COX assay kit selective for COX-1 and COX-2. **(B)** Cell-free supernatants from vehicle or indomethacin treated cells were collected at indicated timepoints post-IR and PGE_2_ secretion was measured with a PGE_2_ enzyme-linked immunosorbent assay. **(A,B)** Graphs represent the mean ± SEM. Each symbol represents an independent sample. Significant differences from UT+ Veh treated cells were determined via **(A)** a student’s *t*-test or **(B)** a one-way ANOVA followed by Bonferroni’s *post hoc* comparisons (**p < 0.01, ****p < 0.0001; ns is not significant).

Indomethacin inhibits both COX-1 and COX-2 functions (Tanaka et al., 2002), but has greater selectivity for COX-1 (Warner et al., 1999; Brown et al., 2001). To evaluate whether indomethacin is COX-1- or COX-2-selective in salivary glands, parotid acinar cells were treated with vehicle or indomethacin (25 μM) for 24 h and COX-1 and COX-2 activities were measured. Results indicate that indomethacin preferentially inhibits COX-1 in primary parotid acinar cells (Figure 2A). We have previously shown that there are elevated levels of PGE_2_ secreted from primary parotid gland cells following IR exposure, with lower PGE_2_ levels detected in IR-exposed P2X7R^–/–^ cells that correlate with improved salivary gland function (Gilman et al., 2019). To determine if indomethacin treatment modulates PGE_2_ secretion, primary parotid gland cells were exposed to 5 Gy radiation with or without indomethacin pre-treatment (25 μM). Results indicate that indomethacin significantly reduces PGE_2_ secretion into primary parotid cell-free supernatants at 24–72 h post-IR, whereas PGE_2_ secretion is also inhibited by indomethacin in cells not treated with IR (Figure 2B). These data show that indomethacin functions by inhibiting COX-1 activity and reduces constitutive and radiation-induced production of PGE_2_ in primary parotid gland cells.

### Prostaglandin E_2_ Treatment Activates JNK Signaling in Primary Parotid Gland Cells

**FIGURE 3:**
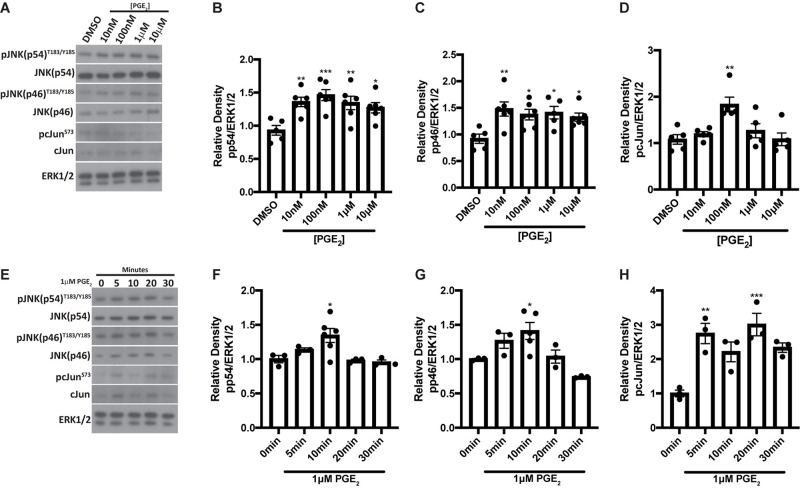
PGE_2_ treatment activates JNK pathways in primary parotid acinar cells. Primary parotid acinar cells were prepared from C57BL/6J mice as described in section “Materials and Methods.” **(A–D)** Dispersed parotid cell aggregates were treated with vehicle (DMSO) or varying doses of PGE_2_ (10 nM, 100 nM, 1 or 10 μM) in serum-free media for 10 min. **(A)** Representative Western blot images of phosphorylated JNK signaling molecules following PGE_2_ treatment at the indicated concentrations. **(B–D)** Densitometry of Western blots represented in panel **(A)**. **(E–H)** Dispersed parotid cell aggregates were treated with vehicle (DMSO) or 1 μM PGE_2_ in serum-free media for 5, 10, 20, or 30 min. **(E)** Representative Western blot images of the kinetics of PGE_2_-mediated phosphorylation of JNK signaling molecules following 1 μM PGE_2_ treatment. **(F–H)** Densitometry of Western blots represented in panel **(E)**. **(A–H)** Immunoblots were generated from cell lysates using anti-phospho-JNK/SAPK (p54/p46)^T183/Y185^ or anti-phospho-c-Jun^S73^ antibodies, which were stripped and re-probed for JNK/SAPK (p54/p46) or c-Jun and then ERK1/2 as a loading control. Densitometry was performed using ImageJ software and protein content was normalized to the average of DMSO-treated cells **(A–D)** or cells treated for 0 min **(E–H)**. Graphs represent the mean ± SEM. Each closed circle represents an independent sample. Significant differences from DMSO-treated cells or cells treated for 0 min were determined via a one-way ANOVA followed by Dunnett’s *post hoc* comparisons (*p < 0.05, **p < 0.01, ***p < 0.001).

We have previously shown that the JNK pathway is activated at day 5 post-IR and promotes radiation-induced compensatory cell proliferation that correlates with the loss of saliva secretion (Grundmann et al., 2010; Hill et al., 2014; Wong et al., 2019). PGE_2_ has been shown to stimulate JNK and c-Jun phosphorylation in human endometrial stromal cells (Zeng et al., 2015) and primary human skin fibroblasts (Arasa et al., 2019). To evaluate whether PGE_2_ activates JNK signaling in salivary glands, primary parotid cell aggregates were treated with varying doses of PGE_2_ for 10 min and phosphorylation of the JNK p54 and p46 isoforms and their downstream target, c-Jun, were assessed via Western analysis. Phosphorylation of both p54 and p46 JNK are enhanced following treatment with all doses of PGE_2_ tested (Figures 3A–C), whereas c-Jun phosphorylation is modestly increased following 100 nM PGE_2_ treatment (Figures 3A,D). To determine kinetic changes in JNK and c-Jun phosphorylation induced by PGE_2_, primary parotid cell aggregates were treated with 1 μM PGE_2_ and phosphorylation of JNK p54 and p46 and c-Jun were assessed after 0, 5, 10, 20, and 30 min. Results indicate that phosphorylation of p54 and p46 JNK are elevated at 10 min following 1 μM PGE_2_ treatment (Figures 3E–G). Interestingly, c-Jun phosphorylation is increased at all timepoints measured, with significantly higher levels observed at 5 and 20 min following PGE_2_ treatment as compared to the 0 timepoint (Figures 3E,H). These data show that PGE_2_ activates the JNK/c-Jun pathway in parotid glands.

### Prostaglandin E_2_ and EP2, 3, and 4 Receptor Agonism Induce Proliferation of Primary Parotid Gland Cells

**FIGURE 4:**
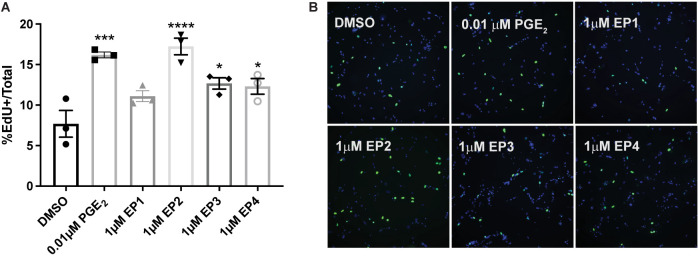
PGE_2_ and EP2, 3 and 4 receptor agonism induce proliferation of primary parotid gland cells. Primary parotid cells were prepared from C57BL/6J mice, serum-starved for 2 h and treated with vehicle (DMSO), PGE_2_ (0.01 μM) or the EP receptor-selective (EP1R-EP4R) agonists for EP1R: 17-phenyl trinor PGE_2_ (1 μM), EP2R: AH13205 (1 μM), EP3R: sulprostone (1 μM) or EP4R: CAY10598 (1 μM). All compounds were solubilized in DMSO, mixed with serum-free primary cell culture media and incubated with cells for 24 h with EdU added during the last hour of treatment to monitor proliferating cells, as described in section “Materials and Methods.” **(A)** Groups were quantified by averaging the number of positive cells out of the total number of cells from 5 fields of view/slide. Graphs represent the mean ± SEM. Each symbol represents an independent sample. Significant differences from DMSO-treated cells were determined via a one-way ANOVA followed by Dunnett’s *post hoc* comparisons (*p < 0.05, ***p < 0.001, ****p < 0.0001). **(B)** Representative fields of view are shown.

It has been shown that JNK activation mediates compensatory proliferation in parotid glands at day 5 post-IR (Wong et al., 2019) and PGE_2_-induced JNK signaling is able to stimulate proliferation of pulmonary epithelial tumor cells (Zhong et al., 2015) and human mesenchymal stem cells (Yun et al., 2011). To evaluate whether PGE_2_ treatment or selective EP receptor (EP1R-EP4R) activation is able to induce cell proliferation in salivary glands, primary parotid gland cells were treated with PGE_2_ or selective agonists for EP1R (17-phenyl trinor PGE_2_; 1 μM), EP2R (AH13205; 1 or 10 μM), EP3R (sulprostone; 0.01 or 1 μM) or EP4R (CAY10598; 1 or 10 μM) for 24 h and EdU incorporation was determined during the last hour of treatment as a measure of cell proliferation. The number of EdU positive cells increases following PGE_2_ treatment (0.01 or 0.03 μM), EP2R agonism (1 or 10 μM AH13205), EP3R agonism (0.01 or 1 μM sulprostone) or EP4R agonism (1 or 10 μM CAY10598) as compared to DMSO-treated cells (Figures 4A,B and Supplementary Figures 1A,B). EP1R agonism (1 μM 17-phenyl trinor PGE_2_) did not increase proliferation levels compared to DMSO-treated controls (Figures 4A,B). These data indicate PGE_2_ increases proliferation in parotid gland cells via either EP2, 3 or 4 receptor activation, which is likely mediating the induction of the compensatory proliferation response seen post-IR (Grundmann et al., 2010; Hill et al., 2014; Wong et al., 2019), downstream of radiation-induced PGE_2_ secretion (Gilman et al., 2019).

### Prostaglandin E_2_ Decreases Amylase Levels and Store-Operated Calcium Entry in Primary Parotid Cells

**FIGURE 5:**
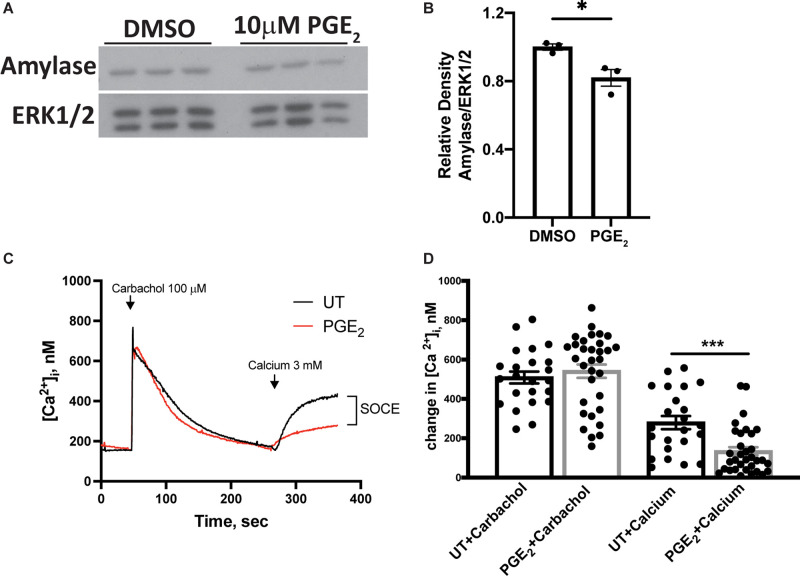
PGE_2_ treatment decreases amylase levels and store-operated calcium entry (SOCE) in primary parotid acinar cells. Primary parotid acinar cells were prepared from C57BL/6J mice as described in section “Materials and Methods.” **(A–D)** Primary parotid gland cells were untreated (UT) or treated with vehicle (DMSO) or PGE_2_ (10 μM) for 24 h. **(A)** Representative Western blot images of changes in amylase levels following PGE_2_ treatment. **(B)** Densitometry of Western blots represented in panel **(A)**. **(A,B)** Immunoblots were prepared from cell lysates with an anti-amylase antibody which were stripped and re-probed for ERK1/2 as a loading control. Densitometry was done using ImageJ software and protein content was normalized to the average of DMSO-treated cells. Graphs represent the mean ± SEM. Each closed circle represents an independent sample. Significant differences from DMSO-treated cells were determined via a student’s *t*-test (*p < 0.05). **(C)** Representative tracings of time-dependent fura-2 fluorescence in mouse primary parotid acinar cells pretreated with or without PGE_2_ (10 μM) for 24 h, stimulated with carbachol (100 μM) in calcium-free media followed by addition of calcium (3 mM) where indicated. **(D)** Peak changes in [Ca^2+^]_*i*_ induced by carbachol and the indicated addition of 3 mM calcium. Values are mean ± SEM of results from 23 to 33 cells from two separate cell preparations as shown with closed circles. Significant differences from untreated cells were determined via a student’s *t*-test (***p < 0.001).

Amylase is a marker for differentiated acinar cells and, following radiation damage, amylase levels are reduced in parotid glands, which correlates with reduced gland function (Grundmann et al., 2010; Hill et al., 2014; Morgan-Bathke et al., 2014). To evaluate whether PGE_2_ influences amylase production in parotid glands, primary parotid cells were treated with 10 μM PGE_2_ for 24 h and amylase levels were determined via Western analysis. Amylase levels are reduced in PGE_2_-treated cells when compared to vehicle-treated cells (Figures 5A,B). To identify other mechanisms by which PGE_2_ mediates alterations in salivary gland function, primary parotid cells were treated with PGE_2_ for 24 h and loaded with the calcium indicating dye, fura-2. Intracellular calcium concentration ([Ca^2+^]_*i*_) was measured for 6 min following carbachol (100 μM) stimulation in media lacking calcium, and 3 mM calcium was added 4.5 min after carbachol stimulation. The peak [Ca^2+^]_*i*_ in parotid gland cells following carbachol stimulation is not different between vehicle and PGE_2_-treated cells, indicating normal functionality of muscarinic type 3 receptors on parotid cells and no effect of PGE_2_ on the [Ca^2+^] in intracellular stores (Figures 5C,D). Following addition of 3 mM calcium into the media, there is reduced re-entry of calcium in PGE_2_-treated cells indicated by a reduction in [Ca^2+^]_*i*_ as compared to vehicle-treated cells, suggesting that PGE_2_ signaling blocks SOCE in parotid glands (Figures 5C,D). Taken together, these data illustrate that PGE_2_ reduces amylase levels and inhibits SOCE in primary parotid cells.

### Indomethacin Treatment Reduces JNK Signaling *in vivo* at Day 8 Post-IR

**FIGURE 6:**
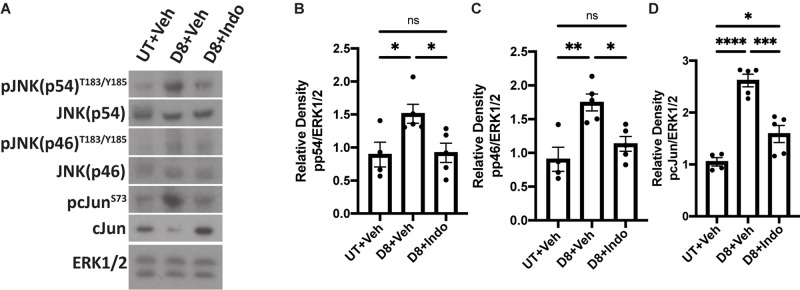
Indomethacin reduces JNK signaling *in vivo* at day 8 post-IR. **(A–D)** C57BL/6J mice were untreated (UT) or received 5 Gy radiation with intraperitoneal injections of vehicle (Veh, saline with 10% ethanol) or indomethacin (Indo, 1 mg/kg body weight) at days 3, 5, and 7 post-IR. Parotid glands were collected at day 8 post-IR. **(A)** Representative Western blot images of phosphorylated JNK signaling molecules following radiation and indomethacin treatments. **(B–D)** Densitometry of Western blots represented in panel **(A)**. **(A–D)** Immunoblots were generated from tissue lysates using antibodies against phospho-JNK/SAPK (p54/p46)^T183/Y185,^ or phospho-c-Jun^S73^, which were stripped and re-probed for JNK/SAPK (p54/p46) or c-Jun and then ERK1/2 as a loading control. Densitometry was performed using ImageJ software and protein content was normalized to the average of UT + Veh-treated mice. Graphs represent the mean ± SEM. Each closed circle represents an independent sample. Significant differences were determined via a one-way ANOVA followed by Bonferroni’s *post hoc* comparisons (*p < 0.05, **p < 0.01, ***p < 0.001, ****p < 0.0001, ns = not significant).

We have previously shown that IR activates JNK signaling 5 days post-IR, which partially regulates the compensatory proliferation response in parotid glands (Wong et al., 2019). It is well described that JNK activation induces c-Jun phosphorylation (Johnson and Nakamura, 2007). To determine if indomethacin treatment modulates JNK signaling *in vivo*, mice with or without 5 Gy IR exposure were injected with vehicle or indomethacin (1 mg/kg body weight) at days 3, 5 and 7 post-IR. Parotid glands were harvested at day 8 and used for immunoblots. Phosphorylation of JNK p54 and p46 are increased in irradiated parotid tissues at day 8 post-IR, a response reduced with indomethacin treatment (Figures 6A–C). Further, phosphorylation of c-Jun that is significantly increased at day 8 post-IR is reversed with indomethacin treatment (Figures 6A,D). These data illustrate that post-IR indomethacin treatment blocks JNK activation in parotid glands, supporting a role for indomethacin in protection from IR-induced salivary gland damage.

### Post-radiation Indomethacin Treatment Enhances Parotid Gland Amylase Levels and Reduces Compensatory Proliferation at Day 30

**FIGURE 7:**
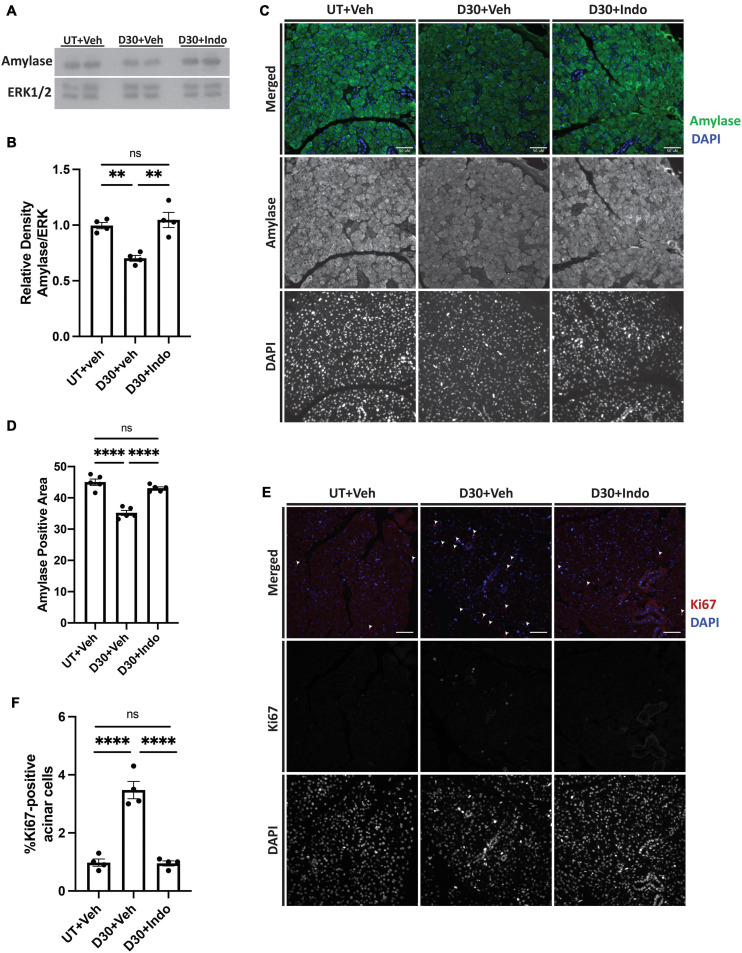
Indomethacin treatment enhances amylase levels and reduces compensatory proliferation in whole parotid glands at day 30 post-IR. **(A,B)** C57BL/6J or (C-F) FVB mice were untreated (UT) or received 5 Gy ionizing radiation (IR) with intraperitoneal injections of vehicle (Veh, saline with 10% ethanol) or indomethacin (Indo, 1 mg/kg body weight) at days 3, 5, and 7 post-IR. Parotid glands were extracted at day 30 post-IR. **(A,B)** Immunoblots were generated from tissue lysates and an anti-amylase antibody, which were stripped and re-probed for ERK1/2 as a loading control. **(A)** Representative Western blot images of changes in amylase levels following radiation and indomethacin treatments. **(B)** Densitometry was performed using ImageJ software and protein content was normalized to the average of the UT + Veh group. **(C–F)** Parotid glands were fixed, sectioned and immunohistochemistry was performed with **(C,D)** an anti-amylase antibody or **(E,F)** an anti-Ki67 antibody, as described in section “Materials and Methods.” **(C)** Representative images of amylase positive area (40× magnification, scale bar: 50 μm). **(D)** Percent positive amylase area was determined using ImageJ software. The graph represents the amylase positive area as a percentage of the total area. **(E)** Representative images of Ki67 positive area (white arrows indicate Ki67+, DAPI+ cells; 40× magnification, scale bar: 50 μm). **(F)** Ki67 positive and total cell numbers were manually counted from 5 fields of view/mouse. The graph represents the Ki67 positive cell number as a percentage of the total cell number. **(B,D,F)** Graphs represent the mean ± SEM. Each closed circle represents an independent sample. Significant differences were determined via a one-way ANOVA followed by Bonferroni’s *post hoc* comparisons (**p < 0.01, ****p < 0.0001; ns = not significant).

To confirm the *in vitro* findings that PGE_2_ decreases amylase secretion, mice were untreated or exposed to 5 Gy radiation with vehicle or indomethacin injections at days 3, 5, and 7 post-IR and parotid glands were collected at day 30, representing a chronic timepoint following damage (Grundmann et al., 2010). Protein was extracted for immunoblot analysis, which shows that total amylase levels are reduced in irradiated, vehicle-injected mice (Figures 7A,B), whereas indomethacin treatment increases amylase levels in irradiated mice to similar levels as unirradiated, vehicle-injected mice (Figures 7A,B). To confirm these findings and evaluate the proportion of amylase producing acinar cells *in vivo*, parotid glands were collected at day 30 for histological analysis and immunohistochemistry was used to determine the area of salivary gland tissue staining positive for amylase (graphed as percent of total area). The proportion of amylase secreting acinar cells is reduced at day 30 in irradiated, vehicle-injected mice, but indomethacin restored amylase levels to those in unirradiated, vehicle-injected mice (Figures 7C,D). To evaluate whether or not indomethacin exerts modulatory effects on other proteins that influence salivary gland secretion, whole tissue homogenates were used for immunoblots to assess levels of aquaporin 5 (AQP5) and muscle, intestine, and stomach expression 1 (MIST1). Irradiated, vehicle-injected mice have reduced levels of AQP5 in parotid gland homogenates when compared to untreated glands, with indomethacin-treated glands having levels that were not statistically different from either group (Supplementary Figures 2A–C). Unexpectedly, MIST1 levels were not significantly different across treatment groups (Supplementary Figures 2A–C). PGE_2_ is able to induce proliferation following EP2-4 activation (Figure 4 and Supplementary Figure 1) and compensatory proliferation that occurs in irradiated salivary glands correlates with loss of function (Grundmann et al., 2010; Hill et al., 2014; Morgan-Bathke et al., 2014). However, the evaluation of indomethacin as a compensatory proliferation modulator post-radiation has not been previously explored. Parotid gland tissue sections were stained for the proliferation marker Ki67 to evaluate differences in compensatory proliferation following radiation in vehicle and indomethacin-treated mice. Ki67 positive cells are significantly increased in irradiated, vehicle-injected mice and reduced with post-radiation indomethacin treatment (Figures 7E,F). These data demonstrate that indomethacin treatment reduces the compensatory proliferation response and increases the concentration of the major enzyme amylase in irradiated parotid glands indicating improved differentiation of acinar cells, which suggests that this pharmaceutical should be further tested for restoration of the normal protein composition of saliva in patients undergoing radiotherapy for HNC.

## Discussion

Wound healing is a highly complex process that requires delicately coordinated signaling for adequate repair to occur. In irradiated salivary glands, there is conflicting data regarding induction of inflammatory responses (Marmary et al., 2016; Lombaert et al., 2020; Zhao et al., 2020). However, it is well characterized that excessive cell proliferation occurs, but without adequate production of functional differentiated cells (Grundmann et al., 2010; Hill et al., 2014; Morgan-Bathke et al., 2014), suggesting that inhibition of the pathways responsible for compensatory proliferation may improve salivary gland function post-IR. Indomethacin is an NSAID that inhibits both COX-1 and COX-2 functions, but COX-1 has a 10-fold lower IC_50_ value for indomethacin than COX-2 (COX-1 IC_50_: 13 nM; COX-2 IC_50_: 130 nM) (Warner et al., 1999). These findings are consistent with our data from primary parotid gland cells, where 25 μM indomethacin caused a significant reduction in COX-1 activity after 24 h but had no effect on COX-2 activity (Figure 2A). Our previous studies demonstrated reduced PGE_2_ secretion from primary parotid gland cells of P2X7R^–/–^ compared to wild type mice that correlated with the preservation of salivary gland function post-IR, although the differences observed in expression levels or activity of COX isoforms did not correlate with changes in PGE_2_ secretion (Gilman et al., 2019). Here, we show that indomethacin reduces PGE_2_ production and secretion in both untreated and irradiated primary parotid cells, which correlates with reduced COX-1 activity (Figures 2A,B). PGE_2_ has many physiological roles under homeostatic and inflammatory conditions. However, in the context of radiation damage to salivary glands, the generation of PGE_2_ appears to be detrimental to the cell regenerative response, suggesting that NSAIDs are a possible therapeutic modality for restoring salivary gland function post-IR (Goldberg, 1986; Dennis and Norris, 2015; Gilman and Limesand, 2020).

While suppressing PGE_2_ production appears to be protective for irradiated salivary glands (Figures 1, 2) (Gilman et al., 2019), how PGE_2_ influences salivary gland dysfunction has not been clearly delineated. Previous work on pancreatitis, an inflammatory disorder that is associated with high serum amylase content, found that PGE_2_ treatment blocks amylase secretion from pancreatic acini (Mössner et al., 1991). Furthermore, canines with allograft pancreatic transplants that were treated with dimethyl-PGE_2_ had reduced amylase levels in urine compared to untreated controls, indicative of improved pancreatic function with PGE_2_ (Garvin et al., 1989). Indomethacin treatment has been shown to increase isoproterenol (ISO)-induced amylase secretion from rat parotid gland tissue, where enhanced amylase secretion observed following ISO (0.1 μM) stimulation was abrogated by co-treatment with PGE_2_ (Hata et al., 1990). Here, we show that PGE_2_ treatment of primary parotid gland cells reduces amylase levels (Figures 5A,B) and that post-IR indomethacin treatment of mice increased amylase levels in whole parotid tissue (Figure 7). We also show that although carbachol-induced Ca^2+^ release from intracellular stores was unaffected, PGE_2_ decreases SOCE in primary parotid gland cells (Figures 5C,D). Research on the mechanism whereby PGE_2_ inhibits SOCE is limited, although studies have linked production of PGE_2_ to activation of SOCE (Kuda et al., 2011; Jairaman et al., 2015). One previous study found that PGE_2_ treatment (2 h) modulated T cell receptor activation-induced calcium mobilization and SOCE in T cells (Choudhry et al., 1999), which express all EPR isoforms (EP1-4Rs) (Nataraj et al., 2001). In the current study, we show that PGE_2_ treatment (24 h) of primary mouse parotid cells that express EP1, EP2, EP3, and EP4 receptors (Supplementary Figure 3) had no effect on carbachol-induced release of intracellular stores but did reduce subsequent SOCE (Figures 5C,D). Radiation has been shown to cause a loss of SOCE in salivary gland cells due to STIM1 cleavage through activation of the TRPM2 pathway (Liu et al., 2017), although there have been no reports on interactions between PGE_2_ and its receptors (EP1-EP4) and this pathway. Further investigation of how PGE_2_ modulates amylase production and SOCE in salivary glands is warranted and this information may be applicable to other exocrine organs, such as the pancreas and lacrimal glands.

PGE_2_ has been shown to activate the JNK/c-Jun pathway (Zeng et al., 2015; Arasa et al., 2019) and induces proliferation of pulmonary tumors (Zhong et al., 2015) and human mesenchymal stem cells (Yun et al., 2011). Previous work from our lab indicated that JNK/c-Jun activation leads to compensatory proliferation at day 5 post-IR (Wong et al., 2019) that remains elevated through day 90 (Grundmann et al., 2010) and reducing this dysregulated compensatory proliferation correlates with improved salivary gland function post-IR (Grundmann et al., 2010; Hill et al., 2014; Wong et al., 2019). The mediators responsible for radiation-induced cell proliferation in salivary glands are not well defined. Here, we show that PGE_2_ treatment of primary parotid gland cells activates the JNK/c-Jun pathway (Figure 3) and PGE_2_ or selective EP2, EP3, or EP4 receptor agonists induce cell proliferation (Figure 4), suggesting that PGE_2_ mediates the compensatory proliferation response observed in parotid glands *in vivo*. Further supporting this observation, post-radiation indomethacin treatment significantly reduced JNK pathway activation in parotid glands of mice (Figure 6) and Ki67 positive staining at day 30 (Figures 7E,F), which correlates with the ability of indomethacin to improve salivary gland function in irradiated salivary glands (Figures 1C–F). Taken together, our data suggest that indomethacin treatment is a viable pharmacotherapeutic approach to protect salivary glands from IR-induced damage by blocking PGE_2_ production and its activation of the JNK/c-Jun pathway leading to dysregulated proliferation. Relevantly, excessive PGE_2_ production has been implicated in the progression of cancer (Nakanishi and Rosenberg, 2013). In multiple HNC models there is evidence that increased production of PGE_2_ increases tumor cell proliferation that can be inhibited with NSAID treatment (Zweifel et al., 2002; Pelzmann et al., 2004; Ye et al., 2004). In HNC xenografts with head and neck squamous cell carcinoma-1483 (SCC-1483) cells, increased COX-2-mediated PGE_2_ production was observed and treatment with a COX-2 selective inhibitor or a PGE_2_-neutralizing antibody reduced tumor growth (Zweifel et al., 2002). Interestingly, treatment of oral SCC-25 cells with genistein, celecoxib or indomethacin reduced PGE_2_ production and cell proliferation levels compared to controls (Ye et al., 2004). Lastly, indomethacin treatment of SCC-25 or SCC-9 cell lines reduced cell growth and induced apoptosis *in vitro* (Pelzmann et al., 2004). Therefore, the use of NSAIDs, such as indomethacin, may be a general means to restrict the growth of cells that become dedifferentiated, as is the case with irradiated salivary gland acinar cells and metastatic tumor cells, both of epithelial origin.

## Data Availability Statement

The original contributions presented in the study are included in the article/Supplementary Material, and further inquiries can be directed to the corresponding author/s.

## Ethics Statement

The animal study was reviewed and approved by the University of Arizona Institutional Animal Care and Use Committee.

## Author Contributions

KG curated and visualized data and drafted the manuscript. KG and JC designed and conducted the experiments. KG, KL, and GW obtained funding for completion of this work. All authors conceptualized experiments, edited the manuscript and approved the final version of the manuscript.

## Conflict of Interest

The authors declare that the research was conducted in the absence of any commercial or financial relationships that could be construed as a potential conflict of interest.

## Supplementary Material

Supplementary Figure 1 Additional doses of PGE_2_ and EP2R, EP3R and EP4R agonists induce proliferation of primary parotid gland cells. Primary parotid cells were prepared from C57BL/6J mice, serum-starved for 2 h and treated with vehicle (DMSO), PGE_2_ (0.03 μM), or EP-receptor-selective (EP2R-EP4R) agonists for EP2R: AH13205 (10 μM), EP3R: sulprostone (0.01 μM) or EP4R: CAY10598 (10 μM). All compounds were solubilized in DMSO, mixed with serum-free primary cell culture media and incubated with cells for 24 h with EdU added during the last hour of treatment to monitor proliferating cells, as described in section “Materials and Methods.” **(A)** Groups were quantified by averaging the number of positive cells out of the total number of cells from 5 fields of view/slide. Graphs represent the mean ± SEM. Each symbol represents an independent sample. Significant differences from DMSO-treated cells were determined via a one-way ANOVA followed by Dunnett’s *post hoc* comparisons (^∗^*p* < 0.05, ^∗∗^*p* < 0.01). **(B)** Representative fields of view are shown.

Supplementary Figure 2 The effect of indomethacin treatment on AQP5 and MIST1 levels in irradiated parotid glands. **(A–C)** C57BL/6J mice were untreated (UT) or received 5 Gy ionizing radiation (IR) with intraperitoneal injections of vehicle (Veh, saline with 10% ethanol) or indomethacin (Indo, 1 mg/kg body weight) at days 3, 5, and 7 following IR and parotid glands were extracted at day 30. Immunoblots were generated from tissue lysates using anti-AQP5 or anti-MIST1 antibodies, and blots were stripped and re-probed for β-tubulin as a loading control. **(A)** Representative Western blot of changes in AQP5 and MIST1 levels following radiation and indomethacin treatments. **(B,C)** Densitometry was performed using ImageJ software and protein content was normalized to the average of the UT + Veh group (^∗^*p* < 0.05; ns = not significant).

Supplementary Figure 3 EP1-4 receptors are present in murine parotid glands. Parotid glands were harvested from untreated (UT) C57BL/6J mice and used for immunoblots. The figure contains representative images of immunoblots for EPRs using antibodies against EP1R, EP2R, EP3R, and EP4R, which were stripped and re-probed for ERK1/2 as a loading control.
