# Supplementary figures and images for: Indomethacin Treatment Post-irradiation Improves Mouse Parotid Salivary Gland Function via Modulation of Prostaglandin E2 Signaling
Source: Front Bioeng Biotechnol. 2021 Jul 21;9:697671. doi: 10.3389/fbioe.2021.697671 (PMC8351468; doi:10.3389/fbioe.2021.697671)

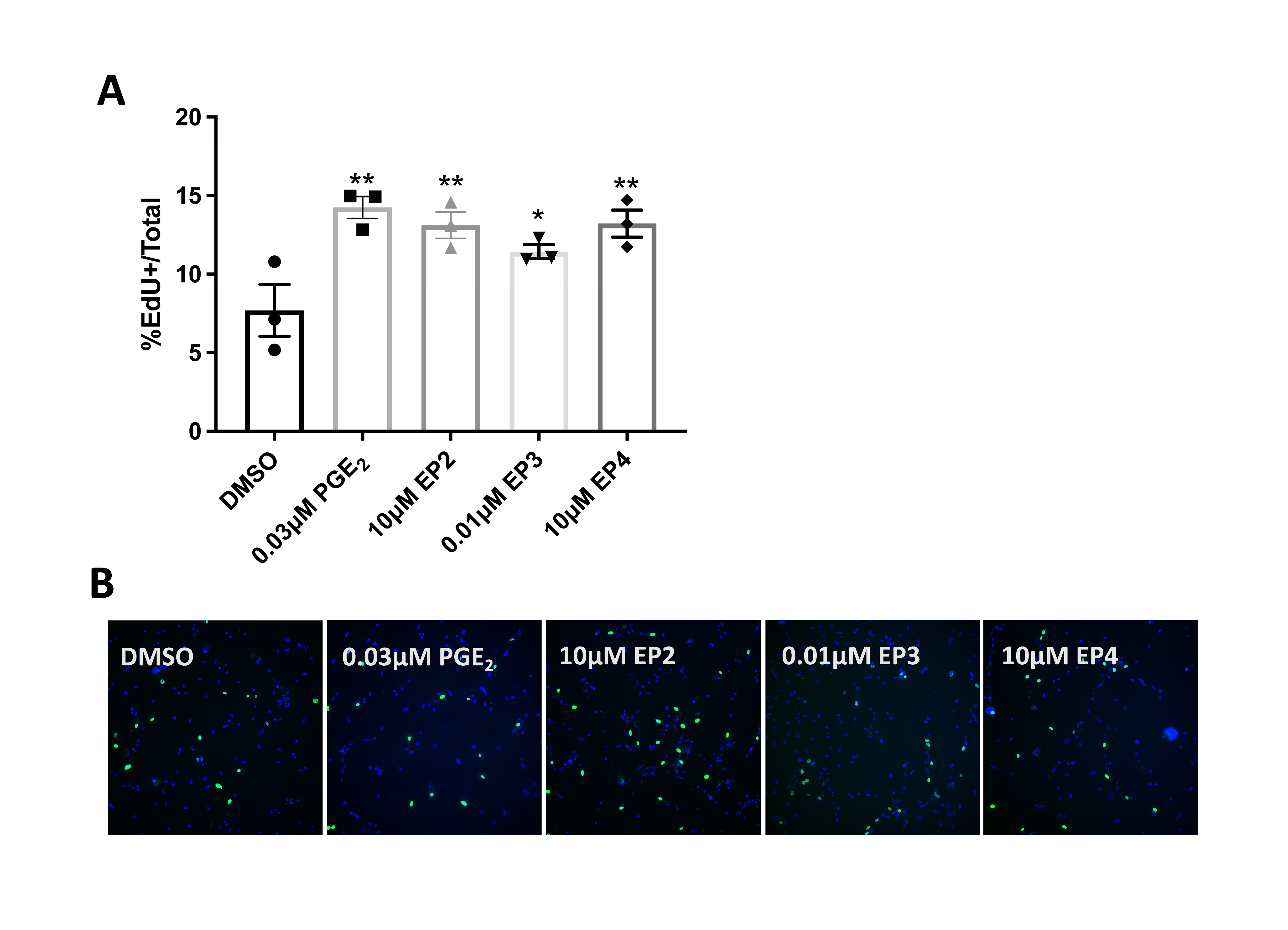

Supplement: Supplementary Figure 1 — Additional doses of PGE2 and EP2R, EP3R and EP4R agonists induce proliferation of primary parotid gland cells. Primary parotid cells were prepared from C57BL/6J mice, serum-starved for 2 h and treated with vehicle (DMSO), PGE2 (0.03 μM), or EP-receptor-selective (EP2R-EP4R) agonists for EP2R: AH13205 (10 μM), EP3R: sulprostone (0.01 μM) or EP4R: CAY10598 (10 μM). All compounds were solubilized in DMSO, mixed with serum-free primary cell culture media and incubated with cells for 24 h with EdU added during the last hour of treatment to monitor proliferating cells, as described in section “Materials and Methods.” (A) Groups were quantified by averaging the number of positive cells out of the total number of cells from 5 fields of view/slide. Graphs represent the mean ± SEM. Each symbol represents an independent sample. Significant differences from DMSO-treated cells were determined via a one-way ANOVA followed by Dunnett’s post hoc comparisons (∗p < 0.05, ∗∗p < 0.01). (B) Representative fields of view are shown. [file Image_1.TIFF]

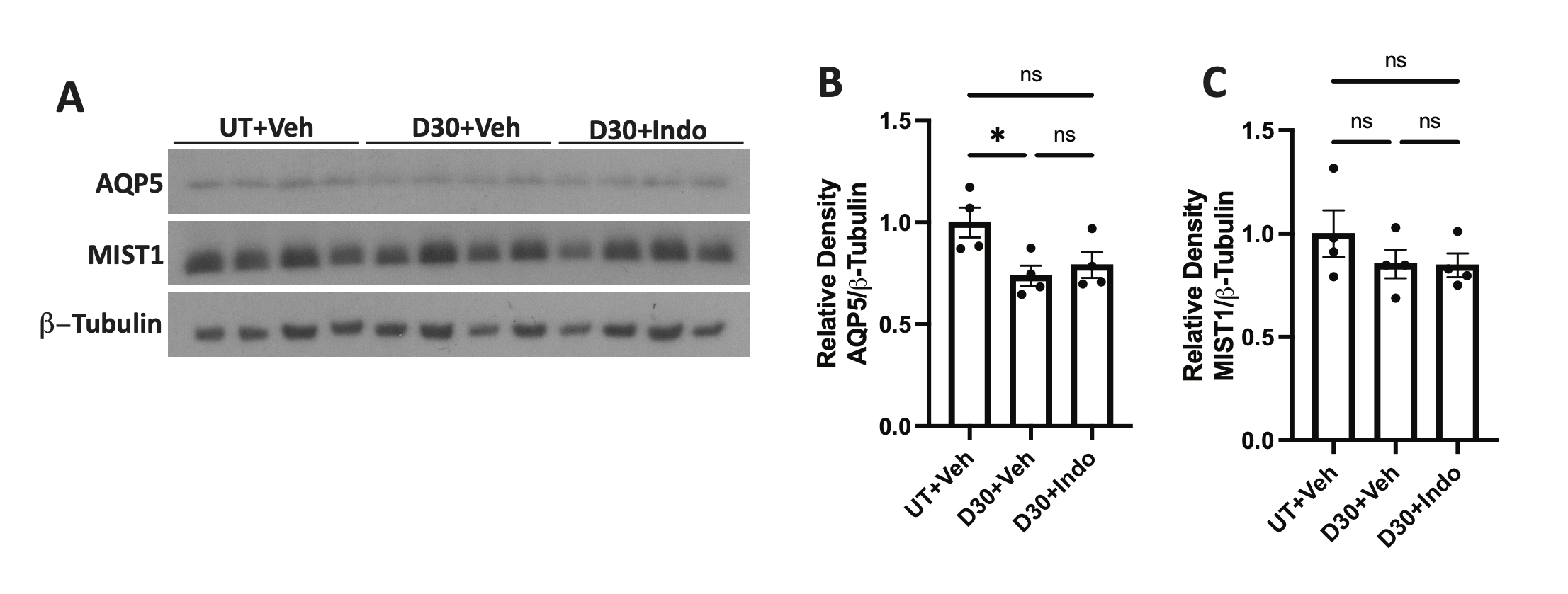

Supplement: Supplementary Figure 2 — The effect of indomethacin treatment on AQP5 and MIST1 levels in irradiated parotid glands. (A–C) C57BL/6J mice were untreated (UT) or received 5 Gy ionizing radiation (IR) with intraperitoneal injections of vehicle (Veh, saline with 10% ethanol) or indomethacin (Indo, 1 mg/kg body weight) at days 3, 5, and 7 following IR and parotid glands were extracted at day 30. Immunoblots were generated from tissue lysates using anti-AQP5 or anti-MIST1 antibodies, and blots were stripped and re-probed for β-tubulin as a loading control. (A) Representative Western blot of changes in AQP5 and MIST1 levels following radiation and indomethacin treatments. (B,C) Densitometry was performed using ImageJ software and protein content was normalized to the average of the UT + Veh group (∗p < 0.05; ns = not significant). [file Image_2.TIFF]

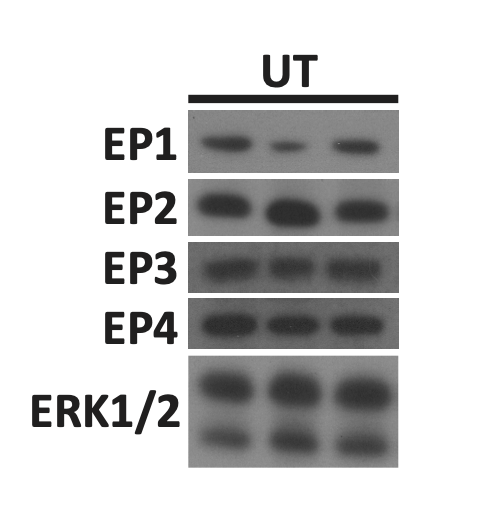

Supplement: Supplementary Figure 3 — EP1-4 receptors are present in murine parotid glands. Parotid glands were harvested from untreated (UT) C57BL/6J mice and used for immunoblots. The figure contains representative images of immunoblots for EPRs using antibodies against EP1R, EP2R, EP3R, and EP4R, which were stripped and re-probed for ERK1/2 as a loading control. [file Image_3.TIFF]
